# Emergency Hysterectomy Following Placental Abruption in a Patient With a History of Substance Abuse: A Case Report

**DOI:** 10.7759/cureus.57997

**Published:** 2024-04-10

**Authors:** Ali Z Ansari, Assem Al Sayed, Srihita Patibandla, Sarthak Kumar, Laasya Patibandla, Rashad Ali

**Affiliations:** 1 Obstetrics and Gynecology, William Carey University College of Osteopathic Medicine, Hattiesburg, USA; 2 Obstetrics and Gynecology, Western University, London, CAN; 3 Obstetrics and Gynecology, South Central Regional Medical Center, Laurel, USA

**Keywords:** maternal-fetal medicine, total abdominal hysterectomy, placental insufficiency, cocaine use, emergency obstetrics, cesarian section, total hysterectomy, disseminated intravascular coagulation (dic), drug and substance abuse, placental abruption

## Abstract

Placental abruption is a serious medical condition that can occur during pregnancy, involving the premature separation of the placenta from the inner uterine wall before childbirth. This detachment often leads to severe bleeding, and if conventional methods prove ineffective in managing the bleeding, a hysterectomy may be deemed necessary to ensure the mother's safety. This case report details the management of a 22-year-old female, gravida IV, para III, who experienced placental abruption during her fourth pregnancy. An emergent cesarean section resulted in severe postpartum hemorrhage and disseminated intravascular coagulation (DIC). Positive drug tests for cocaine and methamphetamines added further complexity, leading to an unplanned hysterectomy for life-saving measures. This case underscores the critical importance of early recognition, multidisciplinary collaboration, and timely intervention in managing obstetric emergencies within the context of substance abuse.

## Introduction

Placental abruption, the complete or partial separation of the placenta from the uterine wall prior to delivery, is one of the major causes of maternal morbidity and perinatal mortality. Various critical complications may rapidly develop as a result of this condition, including obstetric hemorrhage, disseminated intravascular coagulopathy (DIC), renal failure, preterm delivery, asphyxia, stillbirth, and maternal and perinatal death [1]. Thus, recognition of the most common presenting symptoms of placental abruption, including vaginal bleeding, abdominal pain, bloody amniotic fluid, and fetal heart rate abnormalities, and prompt medical intervention is imperative to the health of both maternal and fetal patients [2]. Notably, fetal survival in the setting of placental abruption depends on the gestational age and severity of the abruption [1].

While the pathophysiology of placental abruption is not yet fully understood, various risk factors have been associated with placental abruption, including high parity, low socioeconomic status, hypertension, abdominal trauma, smoking, alcohol use, and substance abuse [1-5]. Substance abuse during pregnancy introduces a multifaceted web of challenges into the realm of obstetric care. Women who engage in substance use, such as cocaine, during pregnancy often exhibit negative alterations in their physiological responses, an elevated risk of obstetric complications, and worse child health outcomes [6]. The coexistence of substance use disorder thus further complicates the intricate medical and surgical management of placental abruption and necessitates careful history-taking despite the emergent nature of the condition.

Several pathological sequelae may arise from substance abuse during pregnancy, potentially leading to a more severe complication that necessitates a hysterectomy and may trigger DIC. The aim of this case report is to shed light on the intricate and multifaceted issues encountered when managing obstetric emergencies in patients with a history of substance abuse and the considerations that should be examined in the process.

## Case presentation

A 22-year-old woman, gravida 4 para 3, with a history of medically managed hypertension, engaged in regular prenatal care from the early stages of her pregnancy. Her previous pregnancies resulted in three uncomplicated deliveries, with the most recent being a cesarean section 13 months ago. In addition, she disclosed a history of substance abuse, including cocaine, marijuana, and heroin use, necessitating immediate enrollment in substance abuse counseling. The patient was sexually active during her pregnancy and reported taking no forms of birth control. Routine ultrasound examinations were conducted during the antenatal period, revealing no abnormalities in fetal development or placental function. The patient received regular antenatal check-ups, including blood pressure monitoring and laboratory investigations, to address the challenges posed by her concurrent medical conditions.

Given the patient's history of a previous cesarean section, hypertension, and substance abuse, discussions regarding the mode of delivery were initiated early in the antenatal period. The patient was informed that a cesarean section was likely, considering the risks associated with a trial of labor after cesarean (TOLAC) and the potential complications associated with her medical history. The patient also had a six-pack-year smoking history, which further added to the complexity of her pregnancy. Despite being informed about the risks associated with substance abuse and smoking during pregnancy, no preventative measures were taken. When the patient presented to the emergency department during her third trimester, her urine drug screen was positive for cocaine and methamphetamines. This posed additional challenges to the healthcare team in managing the potential repercussions for both the mother and the developing fetus.

The patient presented to the hospital at 33 weeks of gestation with heavy vaginal bleeding, abdominal pain, and a rigid abdomen consistent with placental abruption. She was hypotensive and tachycardic at presentation, with a blood pressure of 111/51 mmHg. A physical exam of the patient and bedside ultrasound revealed a firm uterus, occasional contractions, and a fetus with a non-reassuring heart rate. An emergency low-segment transverse cesarean section was promptly performed, and a viable female infant was delivered, weighing 4 pounds and 5 ounces. The patient was discovered to have placenta accreta during the surgery. Immediately following the cesarean section, the patient experienced a hypotensive episode accompanied by profuse vaginal bleeding. Anesthesia providers administered Methergine intramuscularly, and the patient received two units of packed red blood cells (PRBCs). Despite these measures, the patient’s condition continued to deteriorate, and she was diagnosed with likely DIC.

To address the ongoing hemorrhage and coagulopathy, an interdisciplinary surgical team convened in the operating room with the primary objective of stabilizing the patient’s hypothermic and coagulopathic state. Extensive exploration of the pelvic region failed to reveal any overt sources of mechanical bleeding, which prompted the decision to employ pelvic packing as a temporary measure. The procedure involved the meticulous placement of eight laparotomy surgical pads within the pelvis to tamponade ongoing hemorrhage and promote hemostasis. A plastic 1000 drape was utilized to shield the packs and adjacent bowel from direct contact with lap pads and suction components of the dressing. Additional blue towels were layered over this arrangement to enhance pack stability and further safeguard against complications. Furthermore, drains were situated to facilitate appropriate drainage, and the patient’s fluid status was monitored. In the intensive care unit, the patient remained hypotensive and was noted to have subsequent vaginal bleeding after being given Hemabate. Labs revealed a white blood cell (WBC) count of 16,300 thousand, hemoglobin of 7.4 g/dL, platelet count of 65,000, pH of 7.12, pCO_2_ of 51.0 mmHg, PO_2_ of 91 mmHg, oxygen saturation of 94%, magnesium of 1.2 mg/dL, blood urea nitrogen (BUN) of 8 mg/dL, creatinine of 1.13 mg/dL, and glucose of 311 mg/dL. 

The decision for an emergency hysterectomy was made due to the persistence of life-threatening hemorrhage despite pelvic packing. Due to the patient being unresponsive and no family able to be found, the patient was taken to the operating room without consent. In the operating room, a team of surgeons performed a total abdominal hysterectomy. The surgery involved the removal of the uterus and ligation of blood vessels to achieve hemostasis. Intraoperative findings included examination of the uterine arteries, ligaments, and surrounding tissues to identify and address any additional sources of bleeding. Table 1 provides details of the intervention. The goal of the hysterectomy was to definitively control the source of the hemorrhage and stabilize the patient's condition. During the operation, the patient received 20 units of PRBCs with five to six bags of fresh frozen plasma (FFP) and platelets. She also experienced two episodes of cardiopulmonary arrest with only short periods of pulselessness. Three Hemovac drains were placed in the patient's abdomen, which was packed with nine laparotomy pads, two blue towels, and two sheets of 1000 Drape to allow the patient to be transported to the intensive care unit for resuscitation, warming, and correction of coagulopathy.

**Table 1:** Operative phases during emergency hysterectomy

Step	Procedure
Incision and exposure	A midline abdominal incision was made, extending from the pubic bone to the umbilicus, allowing optimal exposure to the pelvic and abdominal structures.
Exploration of the abdominal cavity	Careful exploration of the abdominal cavity was performed to assess the extent of bleeding and identify potential sources. Attention was given to the presence of blood clots and the overall condition of surrounding organs.
Uterine artery ligation	Identification and ligation of the uterine arteries were paramount to halt the primary blood supply to the uterus. This step involved meticulous dissection and ligature placement to ensure effective hemostasis.
Broad ligament dissection	Dissection of the broad ligament was conducted to expose and ligate vessels contributing to the blood supply to the uterus. This step aims to further prevent bleeding and achieve comprehensive hemostasis.
Uterine dissection and isolation	The uterus was carefully dissected from surrounding tissues, isolating it for subsequent removal. This delicate dissection involved separating the uterus from ligaments and nearby structures to facilitate safe extraction.
Ligature placement and control	Additional ligatures were placed as needed to control bleeding from smaller vessels and ensure meticulous hemostasis. Care was taken to avoid damage to adjacent structures during the ligature placement process.
Uterine removal	The dissected uterus was gently lifted from the pelvic cavity. Attention was given to maintaining the integrity of adjacent structures and preventing inadvertent injuries during the removal process.
Closure of the vaginal cuff	Closure of the vaginal cuff was performed to secure the lower end of the hysterectomy site. This step involved suturing the incised portion of the vagina, contributing to the overall closure of the pelvic floor.
Closure of the abdominal incision	The abdominal incision was meticulously closed in layers to ensure optimal wound healing. Attention was given to proper alignment and tension to minimize the risk of postoperative complications.
Irrigation and hemostasis confirmation	Thorough irrigation of the abdominal cavity was conducted to remove any debris or blood clots. The surgical team confirmed hemostasis by assessing for active bleeding and ensuring that all ligatures were secure.

**Figure 1:**
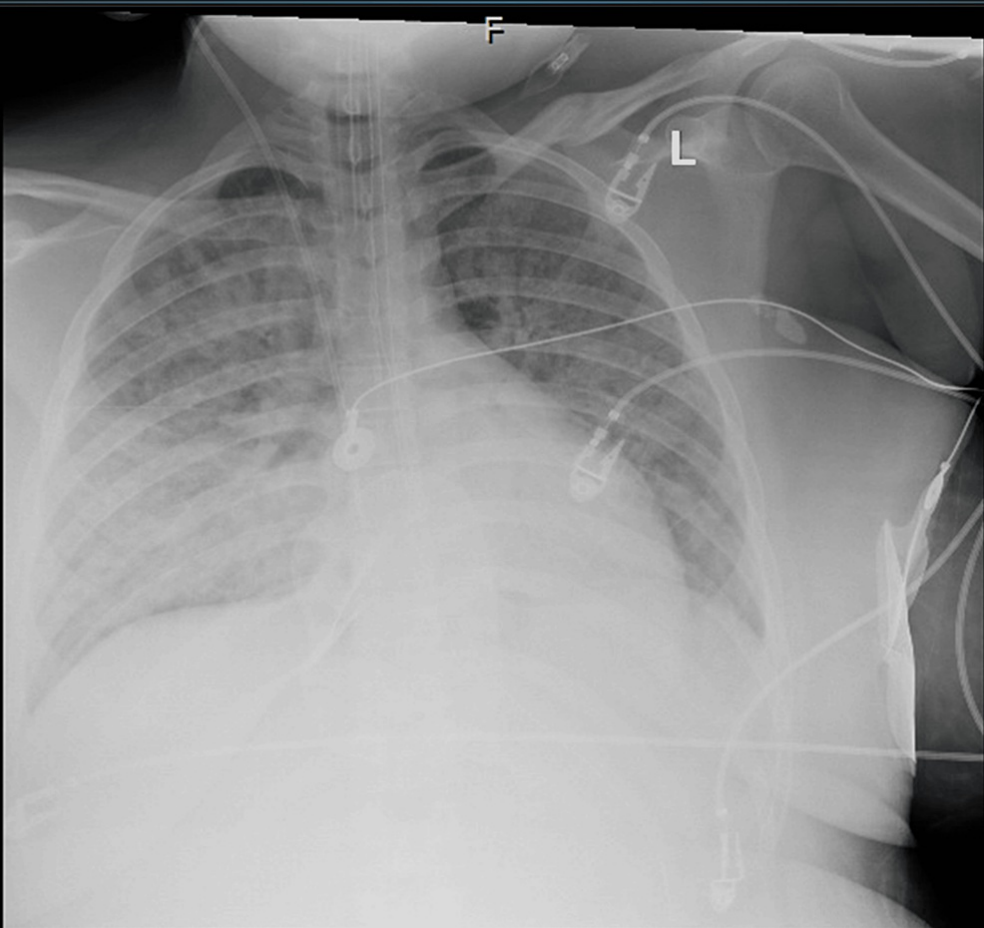
Chest X-ray showing extensive bilateral infiltrates representative of pulmonary edema

Following the operation, the patient remained in a critical condition with an estimated blood loss of over 2 L. The patient was transferred to the intensive care unit, where vigilant monitoring of vital signs and hemodynamic parameters was initiated. The patient remained intubated and ventilated to optimize oxygenation, and the pelvis retained its packed configuration, serving as a temporary measure to effectively manage hemorrhage. A chest X-ray showed pulmonary edema (Figure 1). The patient remained tachycardic with a regular rhythm and a blood pressure of 121/90 mmHg. She had coarse rhonchi with scattered wheezing on lung auscultation. Marked generalized edema was noted in the bilateral lower extremities. Labs revealed sodium of 142 mEq/L, potassium of 3.8 mEq/L, BUN of 13 mg/dL, creatinine of 2.79 mg/dL, aspartate aminotransferase (AST) of 3,781 U/L, alanine aminotransferase (ALT) of 1,359 U/L, WBC of 13.7 thousand, hemoglobin of 8.5 gm/dL, platelets of 109 thousand, PT of 19.3 seconds, INR of 1.6, and aPTT of 30.7 seconds. Arterial blood gas (ABG) readings showed a pH of 7.31 mmHg, pCO_2_ of 35 mmHg, pO_2_ of 64 mmHg, and HCO_3_ of 17.6 mEq/L. The patient had a urine output of 5-10 mK/hour.

**Figure 2:**
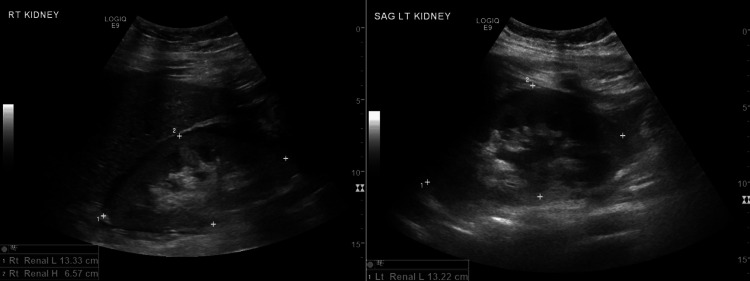
Renal ultrasound showing diffuse increased echogenicity of the renal cortices bilaterally which is consistent with medical renal disease, left mild hydronephrosis, and edema/fluid surrounding both kidneys RT: right, LT: left

She received hourly ABGs in direct ventilator settings and was started on an infusion of bicarbonate for combined metabolic and respiratory acidosis. Blood transfusions were given, magnesium and calcium were replaced as needed, and the patient received pressors to manage hypotension and shock. She was placed on proton-pump inhibitors and sequential compression devices (SCDs) for deep venous thrombosis (DVT) prophylaxis. With continued monitoring, the patient's condition eventually stabilized, and the pulmonary edema and bilateral infiltrates noted in the lungs improved; however, the patient developed acute renal failure with increased creatinine and oliguria, as well as a shock liver, as indicated by her liver function tests. Renal ultrasound revealed increased echogenicity of the renal cortex consistent with medical renal disease, mild left hydronephrosis, and bilateral edema surrounding the kidneys (Figure 2). The patient was taken back to the operating room for the removal of pelvic packing and the passing of a right ureteral stent. However, with continued failure of improvement in renal function, consultations were sought for transfer to an alternate facility to evaluate the patient's need for hemodialysis.

## Discussion

Significant difficulties arise when placental abruption occurs in the setting of drug addiction. The concurrence of placental abruption and substance abuse, especially cocaine and methamphetamine, reveals a complicated web of pathophysiological processes that can have a number of negative impacts on the health of both the mother and the fetus. Being vasoconstrictors, these drugs can cause sudden severe hypertension and impair uterine blood flow in pregnant patients. Placental insufficiency, subsequent to impaired placental perfusion, significantly increases the risk of placental abruption [7]. Furthermore, drugs like cocaine and methamphetamines can cause oxidative stress and an inflammatory reaction in the placental tissue [8]. These processes further harm placental tissues, making the placenta more prone to sudden separation from the uterine wall.

A prompt cesarean section is often required to manage fetal distress and maternal bleeding caused by placental abruption. However, the entry of thromboplastins from the placental injury site into the circulation can cause widespread intravascular activation of the clotting cascade, resulting in DIC [9]. DIC involves the consumption of platelets and clotting proteins, leading to the formation of small blood clots within blood vessels [10]. Obstruction of blood vessels by blood clots leads to organ dysfunction and multisystem organ failure [10]. The interaction between factors that promote and inhibit coagulation is complex, with multiple pathways amplifying the coagulation process. The severe disturbance in blood clotting further complicates the management of placental abruption.

When bleeding becomes uncontrollable, an emergency total abdominal hysterectomy is necessary to save the patient's life [11]. Figure 1 illustrates the decision to perform a hysterectomy. Emergency peripartum hysterectomies are very demanding, and emergent obstetric surgeries are often done in the presence of life-threatening hemorrhage [12]. To add another layer of complexity to the mix, the surgical manipulation required for a hysterectomy may further actively clot factors and fibrinolysis, perpetuating the DIC process. Peripartum hysterectomy often entails significant morbidity and mortality due to the elevated incidence of intraoperative as well as postoperative complications compared to non-obstetric emergencies. The mortality risk associated with peripartum hysterectomy has been shown to be 25 times higher than those performed outside of pregnancy-related cases [13].

**Figure 3:**
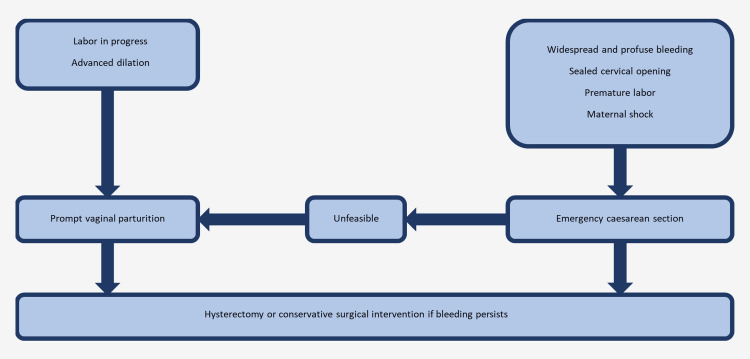
Decision-making algorithm for emergency total abdominal hysterectomy in cases of uncontrollable bleeding This is an original figure created by the authors of this report

The subsequent development of multisystem organ failure, noncardiogenic pulmonary edema, oliguria (low urine output), and acute renal failure is likely caused by multiple factors. The severe hemodynamic disturbances triggered by placental abruption and exacerbated by surgical interventions can initiate systemic inflammatory responses, blood clot formation, and impaired perfusion to vital organs. The inflammatory cascade, initiated by placental abruption and aggravated by substance abuse, can contribute to the dysfunction of the endothelium (the inner lining of blood vessels) and the leakage of fluids into the lungs, resulting in noncardiogenic pulmonary edema [14]. Additionally, the impaired blood flow to the kidneys caused by DIC, compounded by the hemodynamic fluctuations during surgery and the challenges of coagulopathy, can lead to acute renal failure [15]. Oliguria, a common feature of DIC-induced renal dysfunction, indicates reduced kidney filtration capacity, which may require dialysis, as was observed in this case [15].

## Conclusions

In conclusion, the intricate pathophysiological mechanisms contributing to placental abruption in the context of substance abuse reveal a complex interplay involving disruptions in uterine blood flow, coagulation cascades, systemic inflammatory responses, and microvascular thrombosis. This case compellingly illustrates the intricate interplay among maternal health, substance misuse, and obstetric emergencies. It emphasizes the vital need for ongoing research efforts to elucidate the complex mechanistic links, thereby underscoring the inherent intricacy within this clinical context. The findings underscore the necessity for developing targeted strategies for risk reduction and effective management in this challenging clinical scenario. The complexity of the underlying pathophysiology underscores the importance of heightened vigilance, early recognition, and the implementation of comprehensive multidisciplinary care in addressing the unique challenges posed by high-risk pregnancies.
